# C10ORF10/DEPP-mediated ROS accumulation is a critical modulator of FOXO3-induced autophagy

**DOI:** 10.1186/s12943-017-0661-4

**Published:** 2017-05-25

**Authors:** S. Salcher, M. Hermann, U. Kiechl-Kohlendorfer, M. J. Ausserlechner, P. Obexer

**Affiliations:** 1 Department of Pediatrics II, Medical University Innsbruck, Innrain 66, A-6020 Innsbruck, Austria; 2 Department of Pediatrics I, Medical University Innsbruck, Innrain 66, A-6020 Innsbruck, Austria; 3 Tyrolean Cancer Research Institute, Innrain 66, A-6020 Innsbruck, Austria; 4 Department of Anesthesiology and Critical Care Medicine, Medical University Innsbruck, Innsbruck, Austria

**Keywords:** DEPP, FOXO3, Autophagy, Reactive oxygen species

## Abstract

**Background:**

Neuroblastoma is the most common solid tumor in childhood and develops from undifferentiated progenitor cells of the sympathetic nervous system. In neuronal tumor cells DNA-damaging chemotherapeutic agents activate the transcription factor FOXO3 which regulates the formation of reactive oxygen species (ROS) and cell death as well as a longevity program associated with therapy resistance.

We demonstrated before that C10ORF10/DEPP, a transcriptional target of FOXO3, localizes to peroxisomes and mitochondria and impairs cellular ROS detoxification. In the present study, we investigated the impact of FOXO3 and DEPP on the regulation of autophagy. Autophagy serves to reduce oxidative damage as it triggers a self-degradative process for the removal of aggregated or misfolded proteins and damaged organelles.

**Methods:**

The effect of FOXO3 and DEPP on autophagy induction was analyzed using live cell fluorescence microscopy and immunoblot analyses of SH-EP cells transfected with a plasmid for EYFP-LC3 and with siRNAs specific for LC3, respectively. ROS steady-state levels were measured with reduced MitoTrackerRed CM-H2XROS. Cellular apoptosis was analyzed by flow cytometry and the caspase 3/7 assay.

**Results:**

We report for the first time that DEPP induces ROS accumulation and thereby mediates the formation of autophagosomes as inhibition of ROS formation by N-acetyl-cysteine completely blocks autophagy. We further demonstrate that H_2_O_2_-treatment triggers autophagy-induction by FOXO3-mediated DEPP expression. Importantly, knockdown of DEPP was sufficient to efficiently inhibit autophagy-induction under different stress conditions such as serum starvation and genotoxic stress, suggesting that DEPP expression is critical for the initiation of autophagy in neuroblastoma. FOXO3-triggered autophagy partially protects neuroblastoma cells from cell death. Consistent with this concept, we demonstrate that inhibition of autophagy by LC3-knockdown significantly increased etoposide- and doxorubicin-induced apoptosis. These results were also confirmed by the use of the autophagy-inhibitor chloroquine that significantly enhanced the chemotherapeutic effect of etoposide and doxorubicin in neuronal tumor cells.

**Conclusion:**

Targeting FOXO3/DEPP-triggered autophagy is a promising strategy to sensitize neuroblastoma cells to chemotherapy.

**Electronic supplementary material:**

The online version of this article (doi:10.1186/s12943-017-0661-4) contains supplementary material, which is available to authorized users.

## Background

Neuroblastoma, a pediatric tumor of the sympathetic nervous system, covers a broad spectrum of clinical outcome. The majority of neuroblastoma patients with high stage tumors respond poorly to radio- or chemotherapy and show a high relapse rate with a 5-year survival rate of less than 40% [1]. In neuroblastoma, the phosphatidylinositol-3-kinase (PI3K)/protein kinase B (PKB) signaling cascade is frequently deregulated. The transcription factor FOXO3 acts as a critical death-inducing factor downstream of this signaling pathway. Phosphorylation of FOXO3 by PKB induces its association with 14-3-3 proteins and consequently its export from the nucleus and repression of target gene transcription [2–5]. However, oxidative- and genotoxic stress triggers FOXO3-phosphorylation on distinct threonine residues by mammalian sterile 20-like kinase 1 (MST1) and c-Jun N-terminal kinase (JNK) causing its translocation into the nucleus even in the presence of growth factor signaling [6, 7]. In high-stage neuroblastoma tumors nuclear FOXO3 contributes to death resistance under cellular stress conditions that occur during serum starvation or chemotherapy in the tumor tissue [8].

We recently demonstrated that the Decidual Protein induced by Progesterone (C10ORF10/DEPP), a transcriptional target of FOXO3, localizes to peroxisomes and mitochondria and impairs cellular ROS detoxification [9]. Watanabe et al. first described DEPP as a protein that is induced by progesterone in endometrial stromal cells during decidualization [10]. Others found DEPP expression significantly induced by hypoxia in glioblastomas and in human endothelial cells [11, 12] by UV and ionizing radiation [13] or by energy deprivation [14–16], indicating that DEPP might participate in the cellular stress response. Oxidative stress is accompanied by increased ROS accumulation that can lead to mitochondrial dysfunction and cell injury [17]. Under conditions of cellular stress, elevated cellular ROS levels contribute to the induction of autophagy [18, 19]. However, the detailed molecular mechanisms that induce autophagy via ROS are still not well understood (reviewed in [20]).

Autophagy serves to reduce oxidative damage as it triggers a self-degradative process for the removal of aggregated or misfolded proteins and damaged organelles like mitochondria and peroxisomes by sequestration within a double-membrane structure called the autophagosome and successive delivery to lysosomes for degradation [20, 21]. The autophagic process requires a number of autophagy-related genes (ATGs) and is divided into an initiation-, nucleation- and elongation step. To initiate autophagy Unc-51 like kinase 1 (ULK1) phosphorylates Atg13, and focal adhesion kinase family interacting protein of 200 kD (FIP200) [22] which leads to activation of class III phosphatidylinositol-3-kinase (PI3K) and nucleation through BECN1/Beclin-1. Finally Atg5, Atg12, and the microtubule-associated protein light chain 3 (MAP1ALC3/LC3) control the closure of the isolation membrane (reviewed in [23]). In cancer, the tumor-promoting and tumor-suppressing properties of autophagy are still under debate (reviewed in [24]), however, recent studies revealed that the induction of autophagy enhances the resistance of tumors to different anticancer therapies like chemotherapy or radiation therapy [25, 26].

Oehme et al. provided evidence that highly aggressive neuroblastoma cells use autophagy to overcome stress caused by cytotoxic agents, resulting in resistance to chemotherapy [27]. In addition, autophagy regulates the ROS-mediated induction of the cell cycle inhibitor CDKN1A/p21 [28], which controls cellular proliferation, contributes to therapy resistance in cancer [29, 30] and controls phosphorylation of extracellular signal-regulated kinase MAPK1/ERK [31], which is a core regulator of p21 [32, 33].

The FOXO protein family members FOXO1 and FOXO3 modulate autophagy in different cell types [34–39]. FOXO3 transcriptionally controls autophagy by regulating ATG genes and autophagy regulatory genes like LC3 and the GABA[A] receptor-associated protein like 1 (Gabarapl1) [38, 40] and thereby contributes to cell survival under stress conditions [36, 41, 42].

As FOXO3 controls cellular ROS steady-state levels via DEPP expression [9], the present study was designed to investigate the impact of FOXO3 and DEPP on autophagy and associated effects on ROS-mediated cell death as well as therapy resistance in human neuroblastoma.

## Methods

### Cell lines, culture conditions, and reagents

The neuroblastoma cell line STA-NB15 (termed NB15) was isolated at the St. Anna Children’s Hospital (Vienna, Austria) [43]. The neuroblastoma cell line SH-EP was kindly provided by N. Gross, Lausanne, Switzerland [44]. The cell lines were cultured in RPMI 1640 (Lonza, Basel, Switzerland) containing 10% fetal calf serum, 100 U/ml penicillin, 100 μg/ml streptomycin and 2 mM L-glutamine (Gibco BRL, Paisley, GB) at 5% CO_2_ and 37 °C in saturated humidity. Phoenix™ packaging cells for helper-free production of amphotropic retroviruses [45] and HEK293T packaging cells for production of lentiviruses were cultured in DMEM (Lonza, Basel, Switzerland). All cells were routinely tested for mycoplasma contamination using the VenorRGeM-mycoplasma detection kit (Minerva Biolabs, Germany). MnTBAP was purchased from Santa Cruz Biotechnology (Santa Cruz Biotechnology, Texas, USA). All other reagents were purchased from Sigma-Aldrich (Vienna, Austria) unless indicated otherwise. For each experiment, mid-log-phase cultures were seeded in fresh medium.

### Production of retroviruses and lentiviruses for infection of neuroblastoma cells

6 × 10^5^ Phoenix™ packaging cells were transfected with 2 μg of retroviral vectors and 1 μg of a plasmid coding for VSV-G protein using Lipofectamine2000. The vectors pLIB-FOXO3(A3)-ER-iresNeo, pQ-tetCMV-SV40Neo, pQ-tetCMV-EGFP-SV40Neo, pQ-tetCMV-DEPP-SV40-Neo, pQ-tetCMV-EYFP-DEPP-SV40-Neo, pLIB-EYFP-LC3-iresPuro, the lentiviral vectors coding for human DEPP-specific shRNA, FOXO3-specific shRNA and the control vector pLKO.1 have been described previously [2, 3, 9, 46] For expression of YFP-tagged LC3, mus-LC3 was amplified from pEYFP-C1-mus-LC3 (kindly donated from Prof. Noboru Mizushima, Tokyo Medical and Dental University). To generate the vector pLIB-EYFP-LC3-iresPuro the PCR-product was inserted into the EcoR1 and BamH1 site of the previously described pLIB-EYFP-MCL1_JAM_-iresPuro [47] thereby replacing Mcl1L_JAM_ by LC3. The tandem-fluorescent pQCXI-Neo-DsRed-LC3-GFP construct and the Lamp1-RFP plasmid were purchased from Addgene (Addgene, Cambridge, GB).

### Genetically modified neuroblastoma cell lines

The cell lines SH-EP/tetCtr, SH-EP/tetEGFP, SH-EP/tetDEPP, SH-EP/tetEYFP-DEPP, SH-EP/FOXO3-shCtr, SH-EP/FOXO3-shDEPP-10, SH-EP/FOXO3-shDEPP-12, SH-EP/FOXO3-shDEPP-13, SH-EP/shCtr, SH-EP/shFOXO3-17, NB15/shCtr, NB15/shDEPP, and NB15/shFOXO3 have been described previously [3, 9, 48]. SH-EP/FOXO3 cells were infected with retroviral pLIB-EYFP-LC3-iresPuro supernatants to generate SH-EP/FOXO3-EYFP-LC3 cells.

### Immunoblotting

Cells were lysed on ice in lysis-buffer (50 mM TRIS/HCl, 1 mM EDTA, 150 mM NaCl, 1% IPEGAL, 0.25% Deoxycholic acid sodium salt) with protease and phosphatase inhibitors. To analyze LC3 conversion the cells were incubated with 10 μg/ml pepstatin A and 10 μg/ml E-64d. Protein concentration was determined using Bradford-Reagent (BioRad Laboratories, Munich, Germany). Total protein samples (20–50 μg/lane) were separated by SDS-PAGE and transferred to nitrocellulose membranes (Amersham Biosciences, Little Chalfont, GB) by a semi-dry blotting device (Hoefer TE70, Amersham Biosciences). Membrane blocking was performed with PBS blocking buffer containing 0.1% Tween20 and 5% nonfat dry milk, incubated with primary antibodies specific for DEPP, LC3, GAPDH (Novus, Littleton, USA), Gabarapl1 (Abcam, Cambridge, UK), CDK6, p62, ERK1/2, phosphorylated pThr202/Tyr204-ERK1/2 (Cell Signaling, Danvers, USA), p21, p27, Cyclin D1 and CDK4 (BD Biosciences, Heidelberg, Germany). After incubation with anti-mouse or anti-rabbit horseradish-peroxidase-conjugated secondary antibodies the blots were analyzed by the enhanced chemiluminescence substrate ECL Select™ (Amersham Biosciences, Little Chalfont, GB) according to the manufacturer’s instructions and detected with the AutoChemiSystem (UVP, Cambridge, GB). Quantification of protein expression was done with the ImageJ 1.48 software.

### Live cell ROS staining

For ROS measurements, cells were grown on ibidi μ-slide 8 well™ slides (Ibidi, Munich, Germany) and incubated with reduced MitoTrackerRed CM-H2XROS (200 nM, Invitrogen, Carlsbad, CA, USA) for 20 min according to the manufacturer’s instructions. Cells were analyzed by live confocal microscopy using an inverted microscope (Zeiss Observer.Z1; Zeiss, Oberkochen, Germany) in combination with a spinning disc confocal system (UltraVIEW VoX; Perkin Elmer, Waltham, MA, USA). All images were acquired using a 63x oil immersion objective.

### Live cell analysis of autophagic cells

Cells were grown on ibidi μ-slide 8 well™ slides (Ibidi, Munich, Germany) and transfected with 1 μg pLIB-EYFP-LC3-iresPuro, pQCXI-Neo-DsRed-LC3-GFP, or Lamp1-RFP using jetPrime reagent according to the manufacturer’s instructions (PeqLab, Erlangen, Germany). Images were collected with an Axiovert200M microscope equipped with filters for EYFP (exitation: BP500/20, emission: BP535/30) and a 63x oil objective (Zeiss, Oberkochen, Germany) or by live confocal microscopy using an inverted microscope (Zeiss Observer.Z1; Zeiss, Oberkochen, Germany) in combination with a spinning disc confocal system (UltraVIEW VoX; Perkin Elmer, Waltham, MA, USA) and a 63x oil immersion objective.

Quantification of EYFP-LC3 dots per cell was done with the ImageJ 1.48 software.

### Quantitative RT-PCR analyses

To quantify DEPP mRNA levels, we designed “real-time” RT-PCR assays, using GAPDH as reference gene. Total RNA was prepared from 5×10^6^ cells using TRIzol™ Reagent (Invitrogen, Carlsbad, USA) according to the manufacturer’s instructions. cDNA was synthesized from 1 μg of total RNA using the Revert H Minus First Strand cDNA Synthesis Kit (Thermo Scientific, Huntsville, USA). Quantitative RT-PCR was performed as described previously [3] using DEPP (forward ACTGTCCCTGCTCATCCATTCTC and reverse AGTCATCCAGGCTAGGAGAGGG), LC3 (forward AGCAGCATCCAACCAAAATC and reverse CTGTGTCCGTTCACCAACAG), Gabarapl1 (forward ATGAAGTTCCAGTACAAGGAGGA and reverse GCTTTTGGAGCCTTCTCTACAAT), and GAPDH-specific oligonucleotides (forward TGTTCGTCATGGGTGTGAACC and reverse GCAGTGATGGCATGGACTGTG). After normalization on GAPDH expression, regulation was calculated between treated and untreated cells.

### Gene-silencing by siRNA

2×10^5^ cells were seeded in 6-well plates and incubated overnight to reach a confluence of 50%. Cells were transfected with 100 nM siRNA oligonucleotides using jetPrime reagent according to the manufacturer’s instructions (PeqLab, Erlangen, Germany). Twenty-four hours after transfection, cells were plated for the different experiments. SignalSilence® LC3B siRNA I and SignalSilence® Control siRNA were purchased form Cell Signaling (Danvers, USA).

### Determination of apoptosis by flow cytometry

Apoptosis was measured by staining the cells with propidium-iodide (PI) and forward/sideward scatter analysis using a CytomicsFC-500 Beckman Coulter. 2×10^5^ cells were harvested and incubated in 500 μl hypotonic PI solution containing 0.1% Triton X-100 for 4 to 6 h at 4 °C. Stained nuclei in the sub-G1 marker window were considered to represent apoptotic cells.

### Caspase 3/7 assay

Caspase 3/7 activity was determined using Caspase-Glo 3/7 assay kit according to the manufacturer’s instructions (Promega, Madison, USA). Briefly, cells were cultured at 80% confluence in 96-wells containing 50 μl media. Lyophilized Caspase-Glo 3/7 substrate was resuspended in Caspase-Glo 3/7 buffer and 50 μl of this reagent were added to each 96-well and incubated at room temperature for 1 hour. Luminescence of each sample was analyzed with a Hidex Sense microplate reader (Hidex, Turku, Finland). Caspase 3/7 activity was calculated between treated and untreated cells.

### Analysis of cell viability and proliferation

Viability of cells was determined with the AlamarBlue assay (AbD Serotec, Kidlington, UK) in a Hidex Sense microplate reader (Hidex, Turku, Finnland) according to the manufacturer’s instructions. Proliferation of cells was analyzed with a BrdU cell proliferation ELISA kit (Abcam, Cambridge, UK) in a Benchmark Microplate Reader (BioRad Laboratories, Munich, Germany) according to the manufacturer’s instructions. The amount of incorporated BrdU was calculated between treated and untreated cells.

### Statistics

Statistical significance of differences between controls and treated cells were calculated using unpaired *t*-test. All statistical analyses were performed using the Graph Pad Prism 6.0 software.

## Results

### DEPP expression induces autophagy in human neuroblastoma cells

**Fig. 1:**
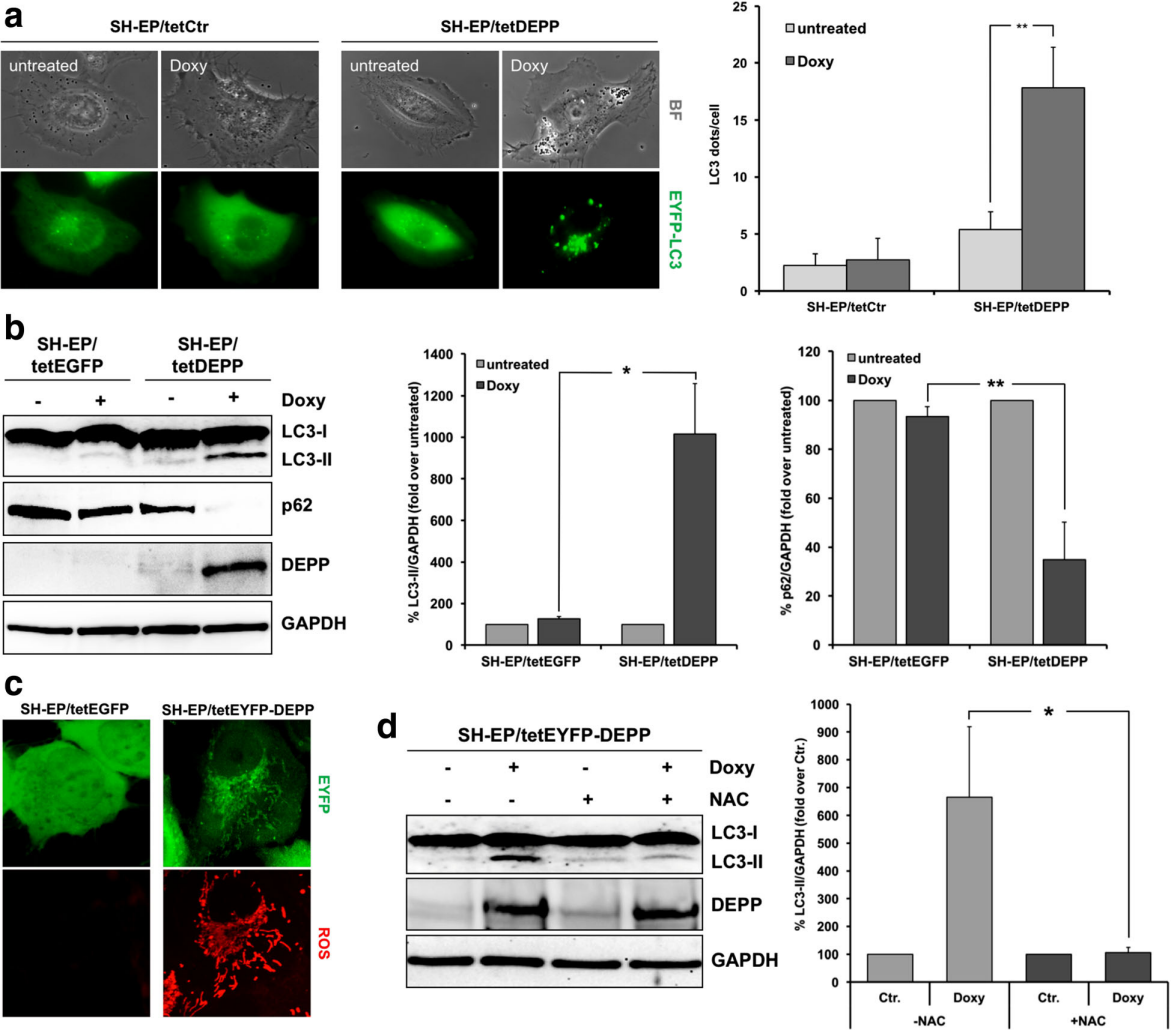
DEPP expression induces autophagy in human neuroblastoma cells. **a** SH-EP/tetCtr and SH-EP/tetDEPP cells were grown on ibidi μ-slide 8 well™ slides and transiently transfected with the pLIB-EYFP-LC3-iresPuro plasmid. Twenty-four hours after transfection the cells were treated with 200 ng/ml doxy for 5 h to induce DEPP expression and analyzed by live cell fluorescence microscopy with an Axiovert200M fluorescence microscope. Autophagy was quantified by counting LC3 dots per cell using the ImageJ 1.48 software. Values are representative results of three independent experiments; statistical analysis was done with the Student’s unpaired *t*-test, **P < 0.025 compared to untreated cells. Values are means ± s.e.m. **b** SH-EP/tetEGFP and SH-EP/tetDEPP cells were treated with 200 ng/ml doxy for 8 h and LC3-I/LC3-II, p62, and DEPP expression were assessed by immunoblot analyses. GAPDH served as loading control. Densitometric analyses were performed with the ImageJ 1.48 software. Untreated cells were set as 100%. Shown are mean values ± s.e.m of three independent experiments; statistical analysis was done with the Student’s unpaired *t*-test, *P < 0.05, **P < 0.025. **c** SH-EP/tetEGFP and SH-EP/tetEYFP-DEPP cells were treated with 200 ng/ml doxy for 4 h. Expression of EGFP and the EYFP-DEPP fusion protein as well as cellular ROS steady state levels were detected by confocal live-cell imaging. **d** SH-EP/tetEYFP-DEPP cells were treated with 200 ng/ml doxy and 5 mM NAC alone and in combination for 8 h. The LC3-I/LC3-II and DEPP expression were determined by immunoblot analyses. GAPDH served as loading control. Densitometric analyses were performed with the ImageJ 1.48 software. Control cells (Ctr.) were set as 100%. Shown are mean values ± s.e.m of three independent experiments; statistical analysis was done with the Student’s unpaired *t*-test, *P < 0.05

To investigate whether DEPP expression induces autophagy in neuroblastoma cells, we measured the formation of LC3-containing autophagosomal vesicles, which is a critical marker for autophagic activity. During autophagy, LC3 undergoes post-translational modifications. The cytosolic LC3-I is lipidated and converted into its active form LC3-II that associates with autophagosomal membranes [49, 50]. To analyze whether DEPP affects LC3, the pLIB-EYFP-LC3-iresPuro plasmid was transiently transfected into SH-EP/tetCtr and SH-EP/tetDEPP cells and ectopic DEPP expression was induced by treatment of the cells with 200 ng/ml doxycycline (doxy). Live-cell imaging analysis revealed an increase in LC3-II punctate in SH-EP/tetDEPP cells due to DEPP expression (Fig. 1a). Quantitative analysis showed a significant increase of the average number of EYFP-LC3 dots per cell from 5.4 ± 1.3 in untreated SH-EP/tetDEPP cells to 17.8 ± 3.5 dots per cell in ectopic DEPP expressing cells (Fig. 1a). These LC3-II punctate primarily represent autophagosomes and are therefore a marker for active cellular autophagy. As shown in SH-EP/tetCtr cells, doxy treatment did not induce LC3-II formation per se, as only the cytoplasmic LC3-I was detectable. In addition to the live-cell imaging experiments we measured the conversion of cytosolic LC3-I into the active, autophagosome membrane-bound form LC3-II on protein level in SH-EP/tetEGFP and SH-EP/tetDEPP cells treated with doxy. By immunoblot analysis using an antibody specific for both, LC3-I- and LC3-II, we found that ectopic DEPP expression induces the conversion of LC3-I into LC3-II in SH-EP/tetDEPP cells (Fig. 1b), which is in line with the live-cell imaging analyses (Fig. 1a). We further analyzed the protein level of p62, also called sequestosome 1 (SQSTM1), which is a marker protein to study autophagic flux. p62 binds directly to ubiquitinated proteins via its UBA-domain and gets degraded during active autophagy [51]. We found p62 protein expression markedly reduced by forced DEPP expression in SH-EP/tetDEPP cells, demonstrating that DEPP induces autophagic flux (Fig. 1b). The impact of DEPP on autophagic flux was monitored by using the autophagy inhibitor chloroquine (CQ), that disrupts the vacuolar H^+^ ATPase responsible for acidifying lysosomes and thereby inhibits autophagosome - lysosome fusion (reviewed in [52]). DEPP-induced LC3-II steady state levels further increased in CQ-treated cells and the repression of the p62 protein level was fully prevented in CQ-treated cells, indicating that autophagic flux is induced by DEPP expression (Additional file 1a). Autophagic flux was further visualized by live-cell fluorescence microscopy of autophagosome and autophagolysosome formation in the presence or absence of CQ using the tandem-fluorescent pQCXI-Neo-DsRed-LC3-GFP construct [53]. These fluorescent analyses with the transiently transfected DsRed-LC3-GFP fusion protein in SH-EP cells are based on the weaker pH stability of GFP compared to dsRed. GFP shows no fluorescence in acidic lysosomes, hence, autophagosomes are labeled yellow (GFP(+)dsRED(+)), while autophagolysosomes are labeled red-only (GFP(−)dsRed(+)). As shown in Additional file 1b ectopic DEPP expression causes the formation of autophagolysosomes, demonstrating active autophagic flux. Inhibition of the autophagosome - lysosome fusion by CQ resulted in predominantly yellow fluorescent vesicles. This suggests that the DEPP-mediated autophagosome formation results from increased *de novo* autophagosome formation and is not the consequence of autophagosome accumulation due to reduced fusion between autophagosomes and lysosomes. We also transfected SH-EP/tetDEPP cells with the Lamp1-RFP construct [54] in combination with the pLIB-EYFP-LC3-iresPuro plasmid to further prove that DEPP expression induces the formation of autophagolysosomes. As shown by live-cell confocal microscopy, the expression of DEPP mediates co-localization of LC3 with LAMP1 in autophagolysosomes in SH-EP/tetDEPP cells treated with doxy, further demonstrating that DEPP induces autophagic flux (Additional file 1c).

We have shown before that DEPP expression affects cellular ROS detoxification capacities in neuroblastoma cells [9]. Thus, we measured ROS steady state levels in SH-EP/tetEGFP and SH-EP/tetEYFP-DEPP cells treated with doxy. Expression of the EYFP-DEPP fusion protein, which localizes to mitochondria and peroxisomes in neuroblastoma cells [9], caused a significant increase of cellular ROS as shown by live-cell imaging analyses using a reduced, non-fluorescent version of the MitoTrackerRed CM-H2XROS that fluoresces upon oxidation (Fig. 1c). As ROS, especially hydrogen peroxide (H_2_O_2_), mediate the induction of autophagy in different cell types (reviewed in [20]), we analyzed whether the DEPP-triggered LC3 conversion is mediated by ROS in neuronal cells. Therefore, we treated SH-EP/tetEYFP-DEPP cells with doxy for 8 h to induce DEPP expression, while ROS formation was inhibited with the ROS scavenger N-acetyl cysteine (NAC). We detected a significant reduction of DEPP-induced LC3 lipidation due to ROS inhibition (Fig. 1d), which suggests that DEPP initiates the formation of autophagosomes by increasing cellular ROS steady-state levels in neuronal cells. In line, DEPP-triggered LC3-II expression was efficiently inhibited using the superoxide dismutase (SOD) mimetic MnTBAP (Additional file 3a). MnTBAP is a potent superoxide anion and peroxynitrite scavenger, but does not scavenge nitric oxide, supporting the notion that intracellular ROS, including superoxides and peroxynitrite, contribute to the induction of DEPP-triggered autophagy.

### FOXO3 induces autophagy through induction of DEPP

As the transcription factor FOXO3 is involved in the modulation of autophagy [37, 38, 55] and DEPP is a transcriptional target of FOXO3 [9], we wondered whether FOXO3 induces autophagy in neuroblastoma cells and whether this process is mediated via DEPP. Therefore, we used SH-EP/FOXO3-shCtr cells that stably express a 4-hydroxy-tamoxifen-inducible (4OHT), PKB-phosphorylation-independent FOXO3(A3)ERtm transgene [2]. DEPP expression was knocked down by lentiviral expression of DEPP-specific shRNAs in these cells [9].

**Fig. 2:**
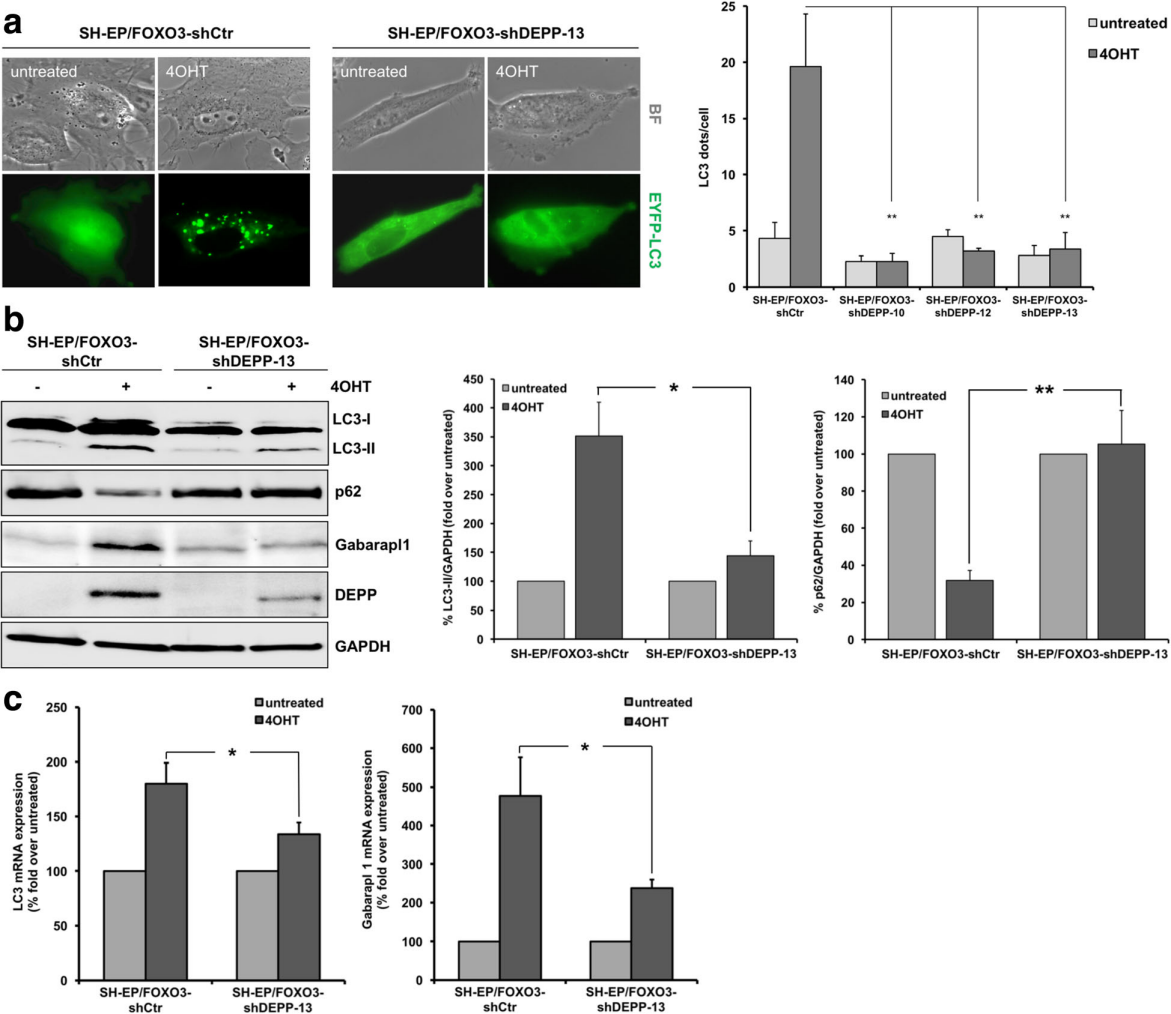
FOXO3-induced autophagy depends on DEPP expression. **a** SH-EP/FOXO3-shCtr and SH-EP/FOXO3-shDEPP-10, −12 and −13 cells were grown on ibidi μ-slide 8 well™ slides and transiently transfected with the pLIB-EYFP-LC3-iresPuro plasmid. Twenty-four hours after transfection the cells were treated with 50 nM 4OHT for 5 h to activate FOXO3 and analyzed by live-cell fluorescence microscopy with an Axiovert200M fluorescence microscope. Autophagy was quantified by counting LC3 dots per cell using the ImageJ 1.48 software. Values are representative results of three independent experiments; statistical analysis was done with the Student’s unpaired *t*-test, **P < 0.025 compared to 4OHT-treated SH-EP/FOXO3-shCtr cells. Values are means ± s.e.m. **b** Immunoblot analyses of LC3-I/LC3-II, Gabarapl1, p62 and DEPP expression of SH-EP/FOXO3-shCtr and SH-EP/FOXO3-shDEPP-13 cells treated with 50 nM 4OHT for 8 h. GAPDH served as loading control. Densitometric analyses of LC3-II and p62 expression relative to GAPDH was done with the ImageJ 1.48 software. Untreated cells were set as 100%. Shown are mean values ± s.e.m of three independent experiments; statistical analysis was done with the Student’s unpaired *t*-test, *P < 0.05, **P < 0.025. **c** SH-EP/FOXO3-shCtr and SH-EP/FOXO3-shDEPP-13 cells were treated with 50 nM 4OHT for 6 h and real time RT-PCR analyses of LC3 and Gabarapl1 expression were performed. Shown are mean values ± s.e.m of three independent experiments; statistical analysis was done with the Student’s unpaired *t*-test, *P < 0.05

To measure LC3-processing we transiently transfected the pLIB-EYFP-LC3-iresPuro construct into SH-EP/FOXO3-shCtr cells and into the three individual SH-EP/FOXO3-shDEPP-10, −12, −13 cell clones. By live-cell imaging analyses we demonstrate that FOXO3 induced the formation of LC3-II positive dots in SH-EP/FOXO3-shCtr cells. The average number of EYFP-LC3 dots per cell significantly increased from 4.3 ± 1.5 to 19.6 ± 4.7 in cells with activated FOXO3 (Fig. 2a). Importantly, DEPP knockdown prevented FOXO3-triggered formation of autophagosomes in all three SH-EP/FOXO-shDEPP cell clones (Fig. 2a). To assess whether also FOXO3 induces autophagic flux the pQCXI-Neo-DsRed-LC3-GFP plasmid was transiently transfected into SH-EP cells. Live-cell fluorescence imaging experiments revealed that FOXO3 triggers the fusion of autophagosomes and lysosomes, indicating active autophagic flux (Additional file 2b). This finding was also reflected by immunoblot analyses of LC3 conversion and p62 expression in SH-EP/FOXO3-shCtr and SH-EP/FOXO3-shDEPP-13 cells treated with 50 nM 4OHT for 8 h (Fig. 2b). FOXO3 induced the expression of LC3-II 4.2-fold over control in SH-EP/FOXO3-shCtr cells, compared to 2.2-fold over control in SH-EP/FOXO3-shDEPP-13 cells. p62 protein expression was reduced in SH-EP/FOXO3-shCtr cells by FOXO3 activation, however p62 protein levels were not altered in SH-EP/FOXO3-shDEPP-13 cells, demonstrating that DEPP expression is necessary to execute FOXO3-regulated autophagy (Fig. 2b). Also Gabarapl1, a transcriptional target of FOXO3 [37], was significantly induced due to FOXO3 activation in SH-EP/FOXO3-shCtr cells, but not in SH-EP/FOXO3-shDEPP-13 cells (Fig. 2b). By LC3-turnover analyses using CQ we could demonstrate that FOXO3-triggered LC3-II expression was further elevated by CQ-treatment. In line, the repression of p62 by FOXO3 was abolished by CQ (Additional file 2a), which supports the notion that FOXO3 induces autophagic flux in neuronal tumor cells.

To determine whether LC3 and Gabarapl1 are also transcriptionally regulated by FOXO3 in neuroblastoma cells we measured the mRNA expression of LC3 and Gabarapl1 in SH-EP/FOXO3-shCtr and SH-EP/FOXO3-shDEPP-13 cells by quantitative RT-PCR analyses. FOXO3 activation caused the upregulation of LC3 (2.2-fold) and Gabarapl1 (4.8-fold) in SH-EP/FOXO3-shCtr cells. However, the FOXO3-triggered induction of these autophagy-related genes was diminished as a consequence of DEPP knockdown in SH-EP/FOXO3-shDEPP-13 cells (Fig. 2c). Together, these data demonstrate that FOXO3 mediates autophagic flux via regulation of DEPP expression in neuronal cancer cells.

### FOXO3 triggered ROS accumulation induces autophagy

We demonstrated before that FOXO3 induces a biphasic ROS accumulation in neuroblastoma cells [3], which is significantly reduced by DEPP knockdown due to an increased capacity to detoxify cellular ROS, especially H_2_O_2_ [9]. Luo et al. found that H_2_O_2_-induced autophagy depends on the induction of intracellular production of ROS [28], raising the question whether FOXO3-mediated autophagy is a consequence of cellular ROS accumulation.

**Fig. 3:**
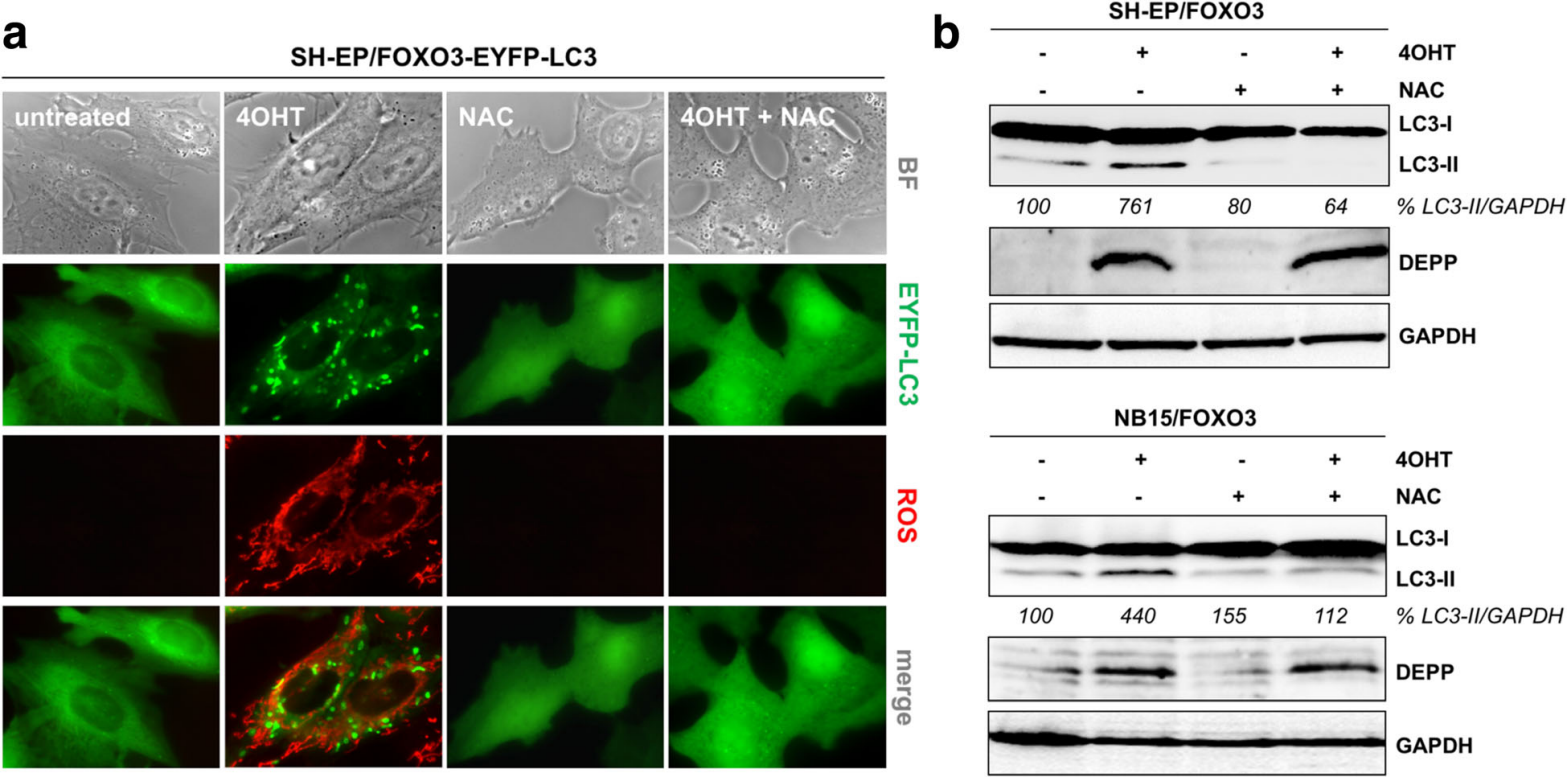
FOXO3 mediated ROS accumulation triggers autophagy. **a** SH-EP/FOXO3-EYFP-LC3 cells were grown on ibidi μ-slide 8 well™ slides. Twenty-four hours after seeding, the cells were treated for 5 h with 100 nM 4OHT to activate FOXO3 and with 7.5 mM NAC to block ROS formation. ROS levels were detected by MitoTrackerRed CM-H2XROS staining. Live-cell fluorescence microscopy was performed with an Axiovert200M fluorescence microscope. **b** Immunoblot analysis of LC3-I/LC3-II expression of SH-EP/FOXO3 and NB15/FOXO3 cells treated with 50 nM 4OHT as well as with 5 mM NAC alone or in combination for 6 h. GAPDH served as loading control. Densitometric analysis of LC3-II expression relative to GAPDH was done with the ImageJ 1.48 software. Untreated cells were set as 100%

We therefore treated SH-EP/FOXO3-EYFP-LC3 cells with 4OHT alone to induce FOXO3 or in combination with the ROS scavenger NAC and measured the accumulation of cellular ROS as well as LC3 dots via live-cell imaging analyses. As shown in Fig. 3a FOXO3 triggers cellular ROS accumulation and formation of LC3 positive vesicles in SH-EP/FOXO3-EYFP-LC3 cells. The ROS scavenger NAC prevented FOXO3-mediated ROS accumulation and in consequence also the formation of autophagosomes (Fig. 3a).

To further prove that FOXO3 triggers autophagy via induction of ROS in neuroblastoma cells we performed immunoblot analysis on LC3 conversion in SH-EP/FOXO3 and NB15/FOXO3 cells treated with 4OHT and with NAC to scavenge ROS. NAC-treatment prevented the FOXO3-mediated LC3 conversion in both cell lines (Fig. 3b). In line, also MnTBAP efficiently abolished FOXO3-mediated LC3-II expression (Additional file 3b), indicating that FOXO3-mediated ROS accumulation triggers autophagy in neuroblastoma cells.

### Cellular stress induces DEPP expression and autophagy

**Fig. 4:**
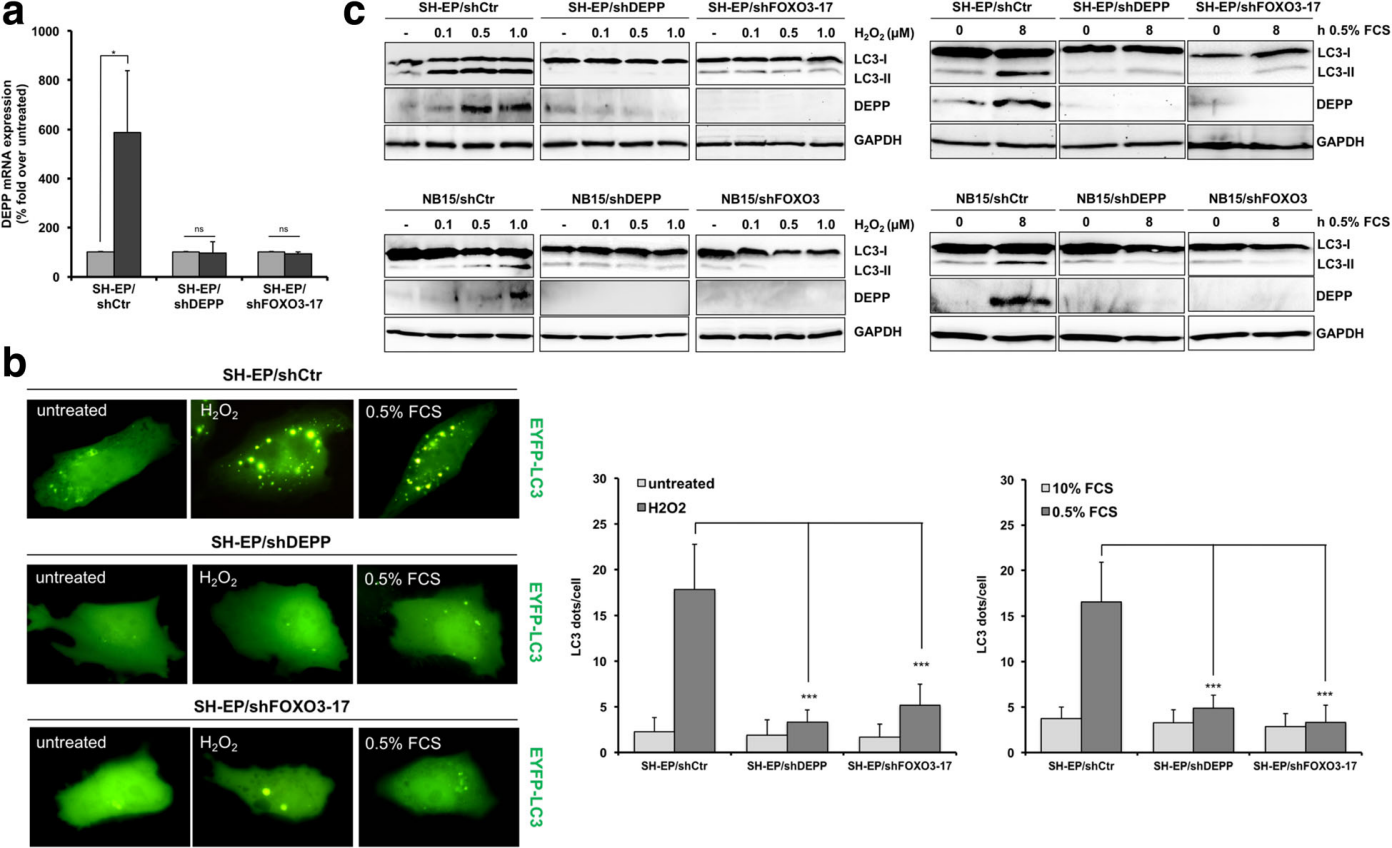
Oxidative stress activates FOXO3/DEPP and thereby triggers autophagy. **a** SH-EP/shCtr, SH-EP/shDEPP, and SH-EP/shFOXO3-17 cells were treated with 1 μM H_2_O_2_ for 1.5 h and real time RT-PCR analyses of DEPP expression were performed. Statistical analysis was done with the Student’s unpaired *t*-test, *P < 0.05 compared to untreated cells. Shown are mean values ± s.e.m of three independent experiments, each performed in triplicates. **b** SH-EP/shCtr, SH-EP/shDEPP as well as SH-EP/shFOXO3-17 cells were grown on ibidi μ-slide 8 well™ slides and transiently transfected with the pLIB-EYFP-LC3-iresPuro plasmid. Twenty-four hours after transfection the cells were treated with 1 μM H_2_O_2_ for 2.5 h or cultured under low serum conditions (0.5% FCS) for 8 h and analyzed by live-cell fluorescence microscopy with an Axiovert200M fluorescence microscope. Autophagy was quantified by counting LC3 dots per cell using the ImageJ 1.48 software. Values are representative results of three independent experiments; statistical analysis was done with the Student’s unpaired *t*-test, ***P < 0.01 compared to untreated controls. Values are means ± s.e.m. **c** Immunoblot analyses of LC3-I/LC3-II and DEPP expression of SH-EP/shCtr, SH-EP/shDEPP, and SH-EP/-shFOXO3-17 as well as NB15/shCtr, NB15/shDEPP, and NB15/shFOXO3 cells treated with the indicated concentrations of H_2_O_2_ for 6 h (upper panel) or cultured at low serum conditions (0.5% FCS) for 8 h (lower panel). GAPDH served as loading control

In a previous study we showed that cellular stress triggered by starvation induces the expression of DEPP in neuronal cells [9]. Stepp et al. found that acute hypoxic stress significantly elevates DEPP mRNA levels in the murine brain and kidney [56], which indicates that different forms of cellular stress lead to DEPP induction. Oxidative stress causes relocalization of FOXO3 from the cytoplasm into the nucleus and activates FOXO3 target genes such as the BH3-only protein BCL2L11/BIM [57, 58]. We therefore analyzed whether oxidative stress affects the expression of DEPP and thereby the induction of autophagy via FOXO3 in neuroblastoma cells. DEPP mRNA expression was significantly elevated in SH-EP/shCtr cells due to oxidative stress triggered by H_2_O_2_-treatment as analyzed by quantitative real time RT-PCR analyses (Fig. 4a). To determine whether the H_2_O_2_-induced DEPP-regulation is mediated by FOXO3 we also performed quantitative RT-PCR analyses of SH-EP/shFOXO3-17 cells. In the single cell clone SH-EP/shFOXO3-17 the FOXO3 expression is silenced by a short hairpin RNA directed against FOXO3. Knockdown of FOXO3 inhibited DEPP regulation by H_2_O_2_, suggesting that FOXO3 is essential for oxidative stress-induced DEPP expression in neuronal cells (Fig. 4a).

To assess, whether cellular stress induces autophagy by activating FOXO3 and DEPP, we transiently transfected the pLIB-EYFP-LC3-iresPuro plasmid into SH-EP/shCtr, SH-EP/shDEPP as well as SH-EP/shFOXO3-17 cells and performed live-cell imaging analyses. Both, H_2_O_2_-treatment and serum starvation (media supplemented with 0.5% FCS) induced the formation of LC3-II punctate in SH-EP/shCtr cells. Importantly, knockdown of DEPP as well as of FOXO3 efficiently impaired the formation of autophagosomes under cellular stress conditions (Fig. 4b). Also immunoblot analyses revealed that H_2_O_2_-treatmentand starvation-triggered autophagy depends on the expression of DEPP and FOXO3 in neuronal cells. We found a marked increase in LC3 conversion during H_2_O_2_-treatment and serum starvation in SH-EP/shCtr and NB15/shCtr cells (Fig. 4c). Knockdown of DEPP prevented LC3 conversion under stress conditions in both neuroblastoma cell lines. Consistently, knockdown of FOXO3 impaired both stress-triggered LC3 lipidation and induction of DEPP expression in SH-EP/shFOXO3-17 as well as in NB15/shFOXO3 cells (Fig. 4c).

Taken together, these results indicate that oxidative and metabolic stress induce the expression of DEPP via FOXO3 and thereby cause the formation of autophagosomes. Importantly, inhibition of the FOXO3/DEPP axis is sufficient to efficiently abrogate the induction of autophagy during cellular stress conditions in neuronal cells.

### DEPP reduces cellular proliferation through LC3-mediated ERK phosphorylation and upregulation of the cell cycle inhibitor p21

**Fig. 5:**
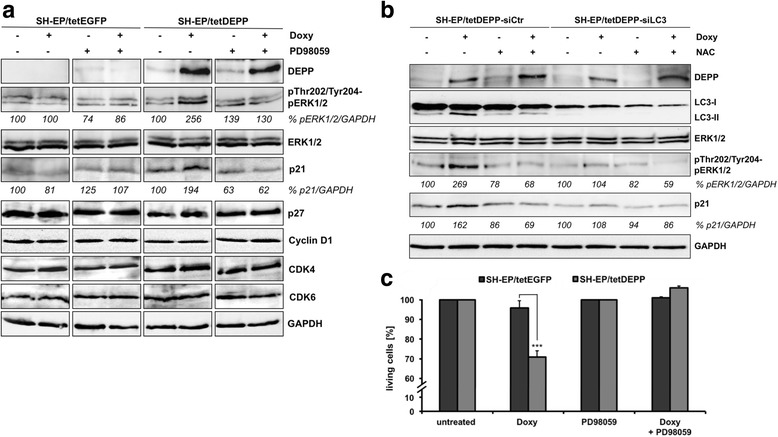
DEPP impairs cellular proliferation through ROS/LC3-mediated ERK1/2 phosphorylation and p21 upregulation. **a** SH-EP/tetEGFP and SH-EP/tetDEPP cells were cultured with 200 ng/ml doxy alone or in combination with 5 μM PD98059 for 24 h and the levels of DEPP, phosphorylated pThr202/Tyr204-ERK1/2, ERK1/2, p21, p27, Cyclin D1, CDK4 and CDK6 were determined by immunoblot analyses. GAPDH served as loading control. Densitometric analysis of pThr202/Tyr204-ERK1/2 relative to ERK1/2 expression and p21 relative to GAPDH was done with the ImageJ 1.48 software. Untreated cells were set as 100%. **b** DEPP, LC3-I/LC3-II, phosphorylated pThr202/Tyr204-ERK1/2, ERK1/2, and p21 expression were assessed by immunoblot analyses of SH-EP/tetDEPP cells transiently transfected with siCtr and siLC3 oligonucleotides and afterwards treated with 200 ng/ml doxy and 5 mM NAC alone or in combination for 24 h. Densitometric analysis of pThr202/Tyr204-ERK1/2 relative to ERK1/2 expression and p21 relative to GAPDH was done with the ImageJ 1.48 software. Untreated cells were set as 100%. **c** The AlamarBlue viability assay was used to quantify the number of living cells. SH-EP/tetEGFP and SH-EP/tetDEPP cells were analyzed after treatment with 200 ng/ml doxy alone or in combination with 5 μM PD98059 for 24 h. Shown are mean values ± s.e.m. of five independent experiments, each performed in triplicates; statistical analysis was done with the Student’s unpaired *t*-test, ***P < 0.001 compared to corresponding controls

Watanabe et al. demonstrated that DEPP expression contributes to ERK1/2 phosphorylation in HEK293 cells [10]. As DEPP leads to ROS accumulation (Fig. 1c) and intracellular ROS at low concentration act as signal transducing molecules stimulating ERK1/2 activation [59–61], we investigated whether ectopic DEPP expression also affects ERK1/2 expression and phosphorylation in neuroblastoma cells. We found a marked induction of ERK1/2 at the phosphorylation sites threonine-202/tyrosine-204 in SH-EP/tetDEPP cells treated with doxy (Fig. 5a). ERK1/2 steady state expression was not regulated by DEPP (Fig. 5a and b). The ERK1/2 pathway is a core regulator of the cell cycle inhibitor p21 [32, 33, 62]. Luo et al. reported that intracellular ROS mediate H_2_O_2_-induced autophagy, which is followed by upregulation of the cell cycle inhibitor p21 [28]. As DEPP induces autophagy via increased ROS steady state levels and contributes to ERK1/2 phosphorylation, we investigated whether DEPP also regulates the expression of p21 in neuroblastoma cells. Immunoblot analyses of p21 expression in SH-EP/tetDEPP cells revealed an upregulation of p21 triggered by ectopic DEPP expression. In agreement with this observation ERK1/2 inhibition abrogated DEPP-mediated p21 induction as shown by immunoblot analyses of SH-EP/tetEGFP and SH-EP/tetDEPP cells treated with doxy and in combination with PD98059, a highly selective inhibitor of the MAP kinase cascade (Fig. 5a). Other cell cycle-related proteins such as CDKN1B/p27, CCND1/cyclin D1, CDK4 and CDK6 were not affected by ectopic DEPP expression (Fig. 5a).

As the autophagy regulatory gene LC3-II is required for ERK phosphorylation in NIH/3 T3 cells [31] and DEPP-induced autophagy is triggered by ROS accumulation, we wondered whether DEPP mediates ERK phosphorylation and p21 induction via ROS and LC3-II conversion in neuroblastoma cells. By immunoblot analyses of SH-EP/tetDEPP cells transiently transfected with scrambled or LC3-specific siRNAs we found that LC3 knockdown and ROS inhibition by NAC attenuated DEPP-triggered ERK1/2 phosphorylation and p21 induction indicating that DEPP-driven ROS accumulation induces autophagy and thereby ERK1/2 phosphorylation and p21 upregulation (Fig. 5b).

As DEPP modulates the expression of the cell cycle inhibitor p21 we assessed the effect of DEPP on the number of viable cells. The AlamarBlue viability assay (Fig. 5c) indicated a significant reduction of living cells due to DEPP expression in SH-EP/tetDEPP cells. In line, we found that forced DEPP expression decreased the amount of incorporated BrdU into SH-EP/tetDEPP cells, indicating reduced proliferation (Additional file 4). In neuroblastoma, ectopic DEPP expression does not induce apoptosis per se [9], supporting the notion that DEPP inhibits cellular proliferation. Importantly, ERK1/2 inhibition by PD98059 was sufficient to prevent DEPP-triggered reduction of proliferation (Fig. 5c). Together, these results indicate that DEPP impairs proliferation of neuronal tumor cells by modulating LC3 and ERK/p21 signaling.

### Autophagy inhibits FOXO3-induced apoptosis in neuroblastoma cells

**Fig. 6:**
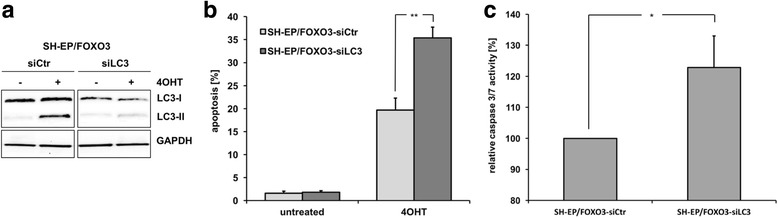
Autophagy protects neuroblastoma cells from FOXO3-triggered apoptosis. **a** SH-EP/FOXO3 cells were transiently transfected with siCtr and siLC3 oligonucleotides and treated afterwards with 50 nM 4OHT for 6 h. LC3-I/LC3-II expression was determined by immunoblot analyses. GAPDH served as loading control. **b** SH-EP/FOXO3-siCtr and SH-EP/FOXO3-siLC3 cells were treated with 50 nM 4OHT for 24 h. PI-FACS analyses were performed to detect apoptotic cells. Shown are mean values ± s.e.m. of three independent experiments; statistical analysis was done with the Student’s unpaired *t*-test, **P < 0.025. **c** The caspase-3/7 activity assay was performed with SH-EP/FOXO3-siCtr and SH-EP/FOXO3-siLC3 cells treated with 50 nM 4OHT for 24 h. The caspase-3/7 activity was calculated between treated and untreated cells. Shown are mean values ± s.e.m. of three independent experiments; statistical analysis was done with the Student’s unpaired *t*-test, *P < 0.05

FOXO3 triggers apoptosis via upregulation of the proapoptotic BH3-only proteins BIM and PMAIP1/NOXA in neuroblastoma cells [2]. FOXO3-mediated induction of BIM disrupts mitochondrial respiration, leading to ROS, which are critical downstream mediators of FOXO3-induced cell death in neuronal cells [3]. However, FOXO3-mediated ROS accumulation simultaneously induces an autophagic program (Fig. 3). As there is evidence for a protective role of autophagy against apoptosis contributing to chemotherapy resistance (reviewed in [24]), we hypothesized that FOXO3 might, besides the regulation of apoptosis, also control a rescue pathway by inducing autophagy in neuroblastoma cells. To study whether autophagy affects FOXO3-induced cell death we inhibited the formation of autophagosomes by transient siRNA-mediated knockdown of LC3 in SH-EP/FOXO3 cells (Fig. 6a). To detect apoptotic cell death, we performed propidium iodide-(PI) FACS-analyses of SH-EP/FOXO3-siCtr and SH-EP/FOXO3-siLC3 cells treated with 4OHT to activate FOXO3. FOXO3-triggered apoptosis was significantly (**P < 0.025) elevated from 19.7% in SH-EP/FOXO3-siCtr cells to 35.4% in SH-EP/FOXO3-siLC3 cells after 24 h of 4OHT-treatment (Fig. 6b). In line with an increase in apoptosis we found caspase-3/7 activities significantly elevated due to inhibition of autophagy in SH-EP/FOXO3-siLC3 cells (Fig. 6c). These results indicate that FOXO3-induced autophagy counteracts FOXO3-triggered apoptosis in neuronal cells.

### Inhibition of autophagy sensitizes neuroblastoma cells to etoposide- and doxorubicin-induced cell death

**Fig. 7:**
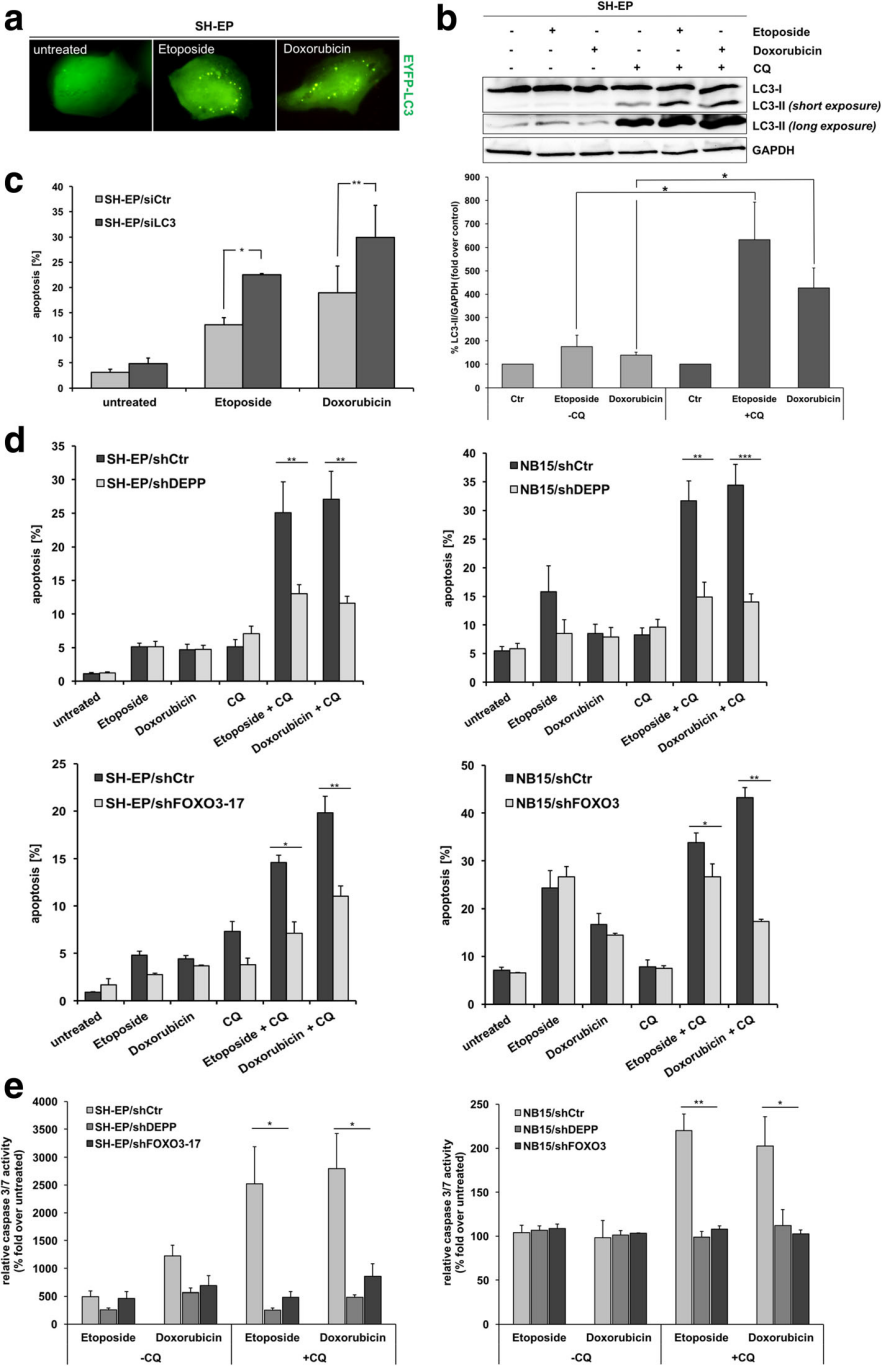
Inhibition of autophagy sensitizes neuroblastoma cells to chemotherapeutic agents. **a** SH-EP cells were grown on ibidi μ-slide 8 well™ slides and transiently transfected with the pLIB-EYFP-LC3-iresPuro plasmid. Twenty-four h after transfection the cells were treated with 20 μg/ml etoposide or with 0.25 μg/ml doxorubicin for 6 h and analyzed by live-cell fluorescence microscopy with an Axiovert200M fluorescence microscope. **b** SH-EP cells were treated with 20 μg/ml etoposide, 0.25 μg/ml doxorubicin, and with 100 μM CQ for 6 h. LC3-I/LC3-II expression was assessed by immunoblot analyses. GAPDH served as loading control. Densitometric analysis of LC3-II relative to GAPDH was done with the ImageJ 1.48 software. Control (Ctr.) cells were set as 100% (long exposure); CQ - treated cells were set as 100% (short exposure). Shown are mean values ± s.e.m of three independent experiments; statistical analysis was done with the Student’s unpaired *t*-test, *P < 0.05. **c** SH-EP cells transiently transfected with siCtr and siLC3 oligonucleotides were treated with 20 μg/ml etoposide and with 0.25 μg/ml doxorubicin for 48 h and PI-FACS analyses were performed to detect apoptotic cells. Shown are mean values ± s.e.m. of three independent experiments; statistical analysis was done with the Student’s unpaired *t*-test, *P < 0.05, **P < 0.025. **d** SH-EP/shCtr, SH-EP/shDEPP (upper panel), and SH-EP/shFOXO3-17 cells (lower panel) were treated with 20 μg/ml etoposide, 0.25 μg/ml doxorubicin, and 100 μM CQ for 24 h. NB15/shCtr and NB15/shDEPP cells (upper panel) were treated with 20 μg/ml etoposide, 0.25 μg/ml doxorubicin, and 50 μM CQ for 48 h. NB15/shCtr and and NB15/shFOXO3 cells (lower panel) were treated with 10 μg/ml etoposide, 0.125 μg/ml doxorubicin, and 50 μM CQ for 48 h. PI-FACS analyses were performed to detect apoptotic cells. Shown are mean values ± s.e.m. of three independent experiments; statistical analysis was done with the Student’s unpaired *t*-test, *P < 0.05, **P < 0.025, ***P < 0.01. **e** The caspase-3/7 activity assay was performed with SH-EP/shCtr, SH-EP/shDEPP, and SH-EP/shFOXO3-17 cells (*left panel*) treated with 20 μg/ml etoposide, 0.25 μg/ml doxorubicin, and 100 μM CQ for 24 h as well as with NB15/shCtr, NB15/shDEPP, and NB15/shFOXO3 cells (*right panel*) treated with 20 μg/ml etoposide, 0.25 μg/ml doxorubicin, and 50 μM CQ for 24 h. The caspase-3/7 activity was calculated between treated and untreated cells. Shown are mean values ± s.e.m. of three independent experiments; statistical analysis was done with the Student’s unpaired *t*-test, *P < 0.05, **P < 0.025

In a previous report, we demonstrated that DNA-damaging chemotherapeutic agents such as etoposide and doxorubicin activate FOXO3 and thereby induce ROS accumulation and apoptosis in neuroblastoma cells [3]. As shown by live-cell imaging analyses, autophagy is induced by etoposide- and doxorubicin-treatment in SH-EP cells (Fig. 7a). Both chemotherapeutic agents induce autophagic flux as the elevated LC3-II expression further increased due to CQ-treatment (Fig. 7b) and the formation of autophagolysosomes can be readily blocked by CQ in SH-EP cells (Additional file 5). To analyze whether autophagy affects cell death induced by chemotherapeutics, we treated SH-EP/siCtr and SH-EP/siLC3 cells with 20 μg/ml etoposide or 0.25 μg/ml doxorubicin for 48 h. Importantly, inhibition of autophagy markedly improved the chemotherapeutic effect of both, etoposide as well as doxorubicin, resulting in a significantly elevated number of apoptotic cells (Fig. 7c).

Inhibition of autophagy by CQ caused a highly significant increase in etoposide- as well as doxorubicin-induced apoptosis and caspase-3/7 activity in SH-EP/shCtr as well as in NB15/shCtr cells (Fig. 7d and e). The increase in apoptosis and caspase-3/7 activity due to autophagy inhibition was significantly reduced by DEPP knockdown, both in SH-EP/shDEPP and in NB15/shDEPP cells (Fig. 7d upper panel and e) as well as by FOXO3 knockdown in SH-EP/shFOXO3-17 and NB15/shFOXO3 cells (Fig. 7d lower panel and e).

These data show that the FOXO3/DEPP-mediated ROS accumulation simultaneously mediates an autophagy and apoptosis program, which counteract each other. The FOXO3/DEPP-triggered autophagy pathway thereby partially protects against chemotherapy-induced cell death and attenuates apoptosis induction in neuroblastoma. The inhibition of autophagic flux by CQ abrogates protective autophagy and might cause additional cellular stress due to accumulation of autophagosomes which sensitizes neuroblastoma cells to chemotherapeutic agent-induced cell death.

## Discussion

In this study, we demonstrate for the first time that the FOXO3-regulated gene DEPP modulates the induction of autophagy in human neuroblastoma. We found that ectopic DEPP expression induces the conversion of LC3-I into its active, membrane-bound form LC3-II, reduces the protein level of p62 (Fig. 1b), and causes the formation of autophagolysosomes as visualized by the colocalization of LC3 with LAMP1 (Additional file 1b and c), demonstrating autophagic flux. Stepp et al. observed that DEPP expression induces autophagy in HEK293 cells [56]. However, the detailed mechanism how DEPP contributes to the activation of autophagy is not described so far. DEPP is induced by different forms of cellular stress [11–16] and impairs the cellular ROS-detoxification capacity [9], which results in increased cellular ROS levels (Fig. 1c). As ROS accumulation contributes to mitochondrial dysfunction, cell injury [17] and to the induction of autophagy (reviewed in [20]), we hypothesized that DEPP might modulate autophagy by increasing ROS levels. Indeed, a marked reduction in DEPP-triggered LC3 conversion was observed due to ROS inhibition by NAC and MnTBAP (Fig. 1d and Additional file 3a).

In neuronal cells, FOXO3 directly regulates DEPP expression by three putative FOXO3 binding sites located on the DEPP promoter [9]. Here we report that FOXO3 leads to autophagic flux via regulation of DEPP as knockdown of DEPP completely abrogated the FOXO3-triggered lipidation of LC3, induction of Gabarapl1, and degradation of p62 (Fig. 2). FOXO3 is a transcriptional activator for autophagy-related genes in different cell types (reviewed in [63]). FOXO3 directly regulates LC3 gene expression in muscle cells [40] and binds to the promoter sequence of Gabarapl1 thereby inducing starvation-mediated autophagy in cardiomyocytes [37]. As DEPP triggers autophagy via intracellular ROS we hypothesized that also FOXO3-mediated LC3 lipidation was ROS- dependent. ROS - inhibition by NAC and MnTBAP prevented FOXO3-triggered autophagy (Fig. 3 and Additional file 3b). In agreement, DEPP knockdown completely inhibited FOXO3-triggered ROS accumulation due to increased cellular ROS detoxification capacities via an increase of the CAT/catalase enzyme activity [9]. When DEPP is expressed, increased cellular ROS steady-state levels might act via a feedback loop and thereby amplify the FOXO3-regulated autophagy program. These data show that FOXO3-triggered autophagy depends on the expression of DEPP and associated ROS accumulation.

Autophagy is described as a cellular rescue pathway during stress conditions as it reduces levels of ROS to protect cell integrity via selective degradation of damaged mitochondria (mitophagy) [23] or peroxisomes (pexophagy) [64]. Li et al. reported that mitochondrial ROS induce autophagy mediated by the AMP-activated protein kinase (AMPK) pathway under starvation conditions [65]. Of note AMPK is a direct regulator of FOXO3 [66]. We recently provided evidence that DEPP expression is regulated via FOXO3 on mRNA and protein level under growth factor withdrawal [9], indicating a possible role of FOXO3/DEPP expression in stress-induced autophagy. DEPP expression is elevated on mRNA- and protein level by oxidative stress triggered by H_2_O_2_-treatment (Fig. 4a and c). Oxidative stress induces nuclear translocation of FOXO transcription factors and functional activation of their target genes [7, 67]. DEPP induction by H_2_O_2_ was strongly reduced by stable FOXO3 knockdown suggesting that DEPP regulation by stress signaling almost exclusively relies on FOXO3 (Fig. 4a). Oxidative and metabolic stress induced the lipidation of LC3 (Fig. 4c) and the formation of autophagosomes (Fig. 4b). Importantly, DEPP- and FOXO3 knockdown experiments revealed that the induction of the FOXO3/DEPP axis by cellular stress is essential to induce autophagy in neuroblastoma (Fig. 4b and c). Also, Warr et al. demonstrated that FOXO3 controls a pro-autophagy gene expression program that specifically directs hematopoietic stem cells to a protective autophagic response upon metabolic stress [42]. However, our studies provide significant new insights into the role of DEPP as a critical link between cellular stress and the induction of autophagy in neuronal tumor cells.

Luo et al. found that intracellular ROS mediate H_2_O_2_-induced autophagy, which is followed by posttranscriptional upregulation of the cell cycle inhibitor p21 and reduced cell proliferation in human fetal lung WI-38 cells [28]. In neuroblastoma, DEPP elevated p21 protein expression (Fig. 5a), which is a result of DEPP-triggered ROS as inhibition of ROS by NAC impaired DEPP-mediated p21 upregulation (Fig. 5b). Of note, autophagy-related proteins including LC3-II stimulate ERK1/2 activity which is a critical upstream regulator of p21 [32]. Martinez-Lopez et al. propose that LC3-II-positive membranes serve as scaffolds for efficient spatial coordination of the Raf-MEK-ERK cascade and thus enable ERK phosphorylation [31]. Several studies have demonstrated that hyperactivation of ERK1/2 causes cell cycle arrest by inducing the expression of p21 (reviewed in [68]), indicating a possible link between DEPP, ERK1/2, and p21 expression. Indeed, conditional induction of DEPP induces ERK1/2 phosphorylation and p21 upregulation (Fig. 5a). Importantly, inhibition of LC3 lipidation by transient expression of LC3-specific siRNA impaired DEPP-triggered ERK1/2 activation as well as p21 induction (Fig. 5b). ERK1/2 inhibition by the MAPK inhibitor PD98059 also prevented p21 upregulation, whereas other cell cycle related proteins were not affected (Fig. 5a). Consistent with the increased p21 expression we found a marked reduction of BrdU incorporation (Additional file 4) and number of viable cells due to increased DEPP expression, which was abolished by ERK1/2 inhibition (Fig. 5c). Together, these results provide first evidence that DEPP-mediated ROS accumulation induces LC3 lipidation, ERK1/2 phosphorylation, and p21 expression, which results in reduced proliferation.

We have shown that FOXO3 causes ROS accumulation, which in turn triggers cell death in neuroblastoma [3]. However, here we report for the first time that FOXO3 simultaneously modulates an autophagy program that partially protects neuroblastoma cells from undergoing apoptosis, suggesting a fragile balance between survival and death controlled by FOXO3. Inhibition of autophagosome formation by LC3 knockdown resulted in a significant increase in FOXO3-triggered activation of caspase 3/7 (Fig. 6c) as well as induction of apoptosis (Fig. 6b). This protective effect of autophagy during stress conditions is in line with several recent studies that describe autophagy as a modulator of resistance to chemotherapy in different types of cancer (reviewed in [24]). Consistent with this concept, the inhibition of autophagy by LC3 knockdown also significantly increased etoposide- and doxorubicin-mediated apoptosis in neuronal cells (Fig. 7c). Both chemotherapeutic agents induce autophagy and autophagic flux in neuroblastoma (Fig. 7b and Additional file 5) similar to what has been published for other cancer types [69, 70]. The idea that autophagic processes are essential for eliminating damaged proteins and organelles to preserve tumor cell life also during chemotherapy was further supported by using the autophagy-inhibitor CQ that significantly enhanced the chemotherapeutic effect of etoposide and doxorubicin in SH-EP and NB15 cells (Fig. 7d and e). Of note, the FDA-approved substance CQ has shown antitumor activity in different cancer types (reviewed in [24]) and might be a useful drug to overcome autophagy-mediated death resistance of neuroblastoma tumors in vivo.

**Fig. 8:**
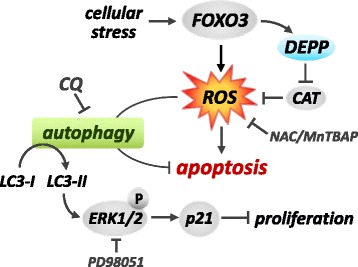
Proposed model for the FOXO3-DEPP-ROS axis in the regulation of life - death balance via autophagy. Cellular stress activates the transcription factor FOXO3 which in turn triggers ROS accumulation and DEPP expression. DEPP represses the cellular ROS detoxification capacity by reducing the CAT/catalase activity and thereby further increases ROS accumulation, which induces both, autophagy and apoptosis. Hence, FOXO3/DEPP modulate an autophagy program via increased cellular ROS to efficiently degrade damaged proteins and organelles and thereby protect neuroblastoma cells from apoptosis. The ROS scavenger NAC and MnTBAP efficiently repress FOXO3/DEPP-triggered autophagy. Repression of DEPP results in reduced ROS levels [9] and consequently neither apoptosis nor autophagy are executed. Inhibition of FOXO3/DEPP-mediated autophagy via CQ or LC3-specific siRNA sensitizes neuroblastoma cells to chemotherapy-induced, apoptotic cell death. DEPP-triggered ROS mediate LC3-lipidation, ERK1/2 phosphorylation, and consequently p21 induction that results in reduced cellular proliferation. Both, LC3 - knockdown and ROS - inhibition by NAC, attenuate DEPP-triggered ERK1/2 phosphorylation. Inhibition of ERK1/2 phosphorylation by PD98051 attenuates DEPP-mediated p21 induction and thereby restores proliferation

The critical role of FOXO3 and DEPP in regulating the life - death balance via autophagy was demonstrated by knockdown of FOXO3 or DEPP, which markedly reduced the chemosensitizing effect of CQ in etoposide- and doxorubicin-treated cells (Fig. 7d and e). We have shown before that DNA-damaging chemotherapeutic agents activate FOXO3/DEPP [3] and that high levels of DEPP reduce the cellular ROS-detoxifying capacity [9], which in turn will lead to cellular damage, but also, as demonstrated in this paper, to increased elimination of damaged proteins and organelles by autophagy (Fig. 8). This divalent function of ROS contributes to keeping the system in balance as long as autophagy is intact and ROS levels remain moderate. When blocking the autophagic elimination pathway by CQ, however, the combustion of the degradation system and the accumulation of damaged proteins and damaged, ROS-producing mitochondria shift the balance to death, which also significantly amplifies the death-inducing effects of chemotherapeutic agents used in neuroblastoma therapy. By this, we discovered an interesting point of attack for therapy and demonstrate that a cheap, FDA-approved compound that has been used decades for the treatment of malaria with moderate side effects can act as very potent chemosensitizer for neuroblastoma therapy.

## Conclusions

Taken together, in this paper we delineate a complex cellular network based on the FOXO3 - DEPP - ROS axis that steers the life - death balance in response to chemotherapy and we reveal that autophagy serves as an essential degradation pathway that can be targeted for chemosensitization by the FDA-approved compound CQ.

## Additional files

Additional file 1: DEPP expression induces autophagic flux. **a** SH-EP/tetEGFP and SH-EP/tetDEPP cells were treated with 200 ng/ml doxy and 100 μM CQ for 8 h. LC3-I/LC3-II and p62 expression were assessed by immunoblot analyses. GAPDH served as loading control. Densitometric analyses were performed with the ImageJ 1.48 software. Control (Ctr.) and CQ-treated cells were set as 100%. Shown are mean values ± s.e.m of three independent experiments; statistical analysis was done with the Student’s unpaired *t*-test, *P < 0.05. **b** SH-EP/tetDEPP cells were grown on ibidi μ-slide 8 well™ slides and transiently transfected with the pQCXI-Neo-DsRed-LC3-GFP plasmid. Forty-eight hours after transfection, the cells were treated with 200 ng/ml doxy and 100 μM CQ for 5 h and analyzed by confocal live-cell imaging. **c** SH-EP/tetDEPP cells were grown on ibidi μ-slide 8 well™ slides and transiently transfected with the pLIB-EYFP-LC3-iresPuro and the Lamp1-RFP plasmid. Forty-eight hours after transfection, the cells were treated with 200 ng/ml doxy and 100 μM CQ for 5 h and analyzed by confocal live-cell imaging. (PDF 1.54mb)

Additional file 2: FOXO3 induces autophagic flux. **a** SH-EP/FOXO3-shCtr and SH-EP/FOXO3-shDEPP-13 cells were treated with 50 nM 4OHT and 100 μM CQ for 8 h. LC3-I/LC3-II and p62 expression were assessed by immunoblot analyses. GAPDH served as loading control. Densitometric analyses were performed with the ImageJ 1.48 software. Control (Ctr.) and CQ-treated cells were set as 100%. Shown are mean values ± s.e.m of three independent experiments; statistical analysis was done with the Student’s unpaired *t*-test, *P < 0.05, **P < 0.025. **b** SH-EP/FOXO3 cells were grown on ibidi μ-slide 8 well™ slides and transiently transfected with the pQCXI-Neo-DsRed-LC3-GFP plasmid. Forty-eight hours after transfection the cells were treated with 50 nM 4OHT and 100 μM CQ for 5 h and analyzed by confocal live-cell imaging. (PDF 1.16mb)

Additional file 3: FOXO3/DEPP-mediated ROS induce LC3 conversion **a** SH-EP/tetEYFP-DEPP cells were pretreated with 100 μM MnTBAP for one hour and incubated with 200 ng/ml doxy and 100 μM MnTBAP alone and in combination for 8 h. The LC3-I/LC3-II and DEPP expression were determined by immunoblot analyses. GAPDH served as loading control. Densitometric analyses were performed with the ImageJ 1.48 software. Untreated cells were set as 100%. **b** Immunoblot analyses of LC3-I/LC3-II expression of SH-EP/FOXO3 cells pretreated with 100 μM MnTBAP and incubated with 50 nM 4OHT and 100 μM MnTBAP alone or in combination for 6 h. GAPDH served as loading control. Densitometric analysis of LC3-II expression relative to GAPDH was done with the ImageJ 1.48 software. Untreated cells were set as 100%. (PDF 255kb)

Additional file 4: Conditional expression of DEPP reduces cellular proliferation. The BrdU cell proliferation ELISA assay was used to quantify the amount of incorporated BrdU during S-phase of proliferating cells. SH-EP/tetEGFP and SH-EP/tetDEPP cells were analyzed for incorporated BrdU after treatment with 200 ng/ml doxy for 48 h. Shown are mean values ± s.e.m. of three independent experiments; statistical analysis was done with the Student’s unpaired *t*-test, **P < 0.025 compared to corresponding controls. (PDF 138kb)

Additional file 5: Etoposide and doxorubicin induce autophagic flux in neuroblastoma cells. SH-EP cells were grown on ibidi μ-slide 8 well™ slides and transiently transfected with the pQCXI-Neo-DsRed-LC3-GFP plasmid. Forty-eight hours after transfection the cells were treated with 20 μg/ml etoposide, 0.25 μg/ml doxorubicin, and with 100 μM CQ for 6 h and analyzed by confocal live-cell imaging. (PDF 904kb)
