# Supplementary figures and images for: C10ORF10/DEPP-mediated ROS accumulation is a critical modulator of FOXO3-induced autophagy
Source: Mol Cancer. 2017 May 25;16:95. doi: 10.1186/s12943-017-0661-4 (PMC5445297; doi:10.1186/s12943-017-0661-4)

a)

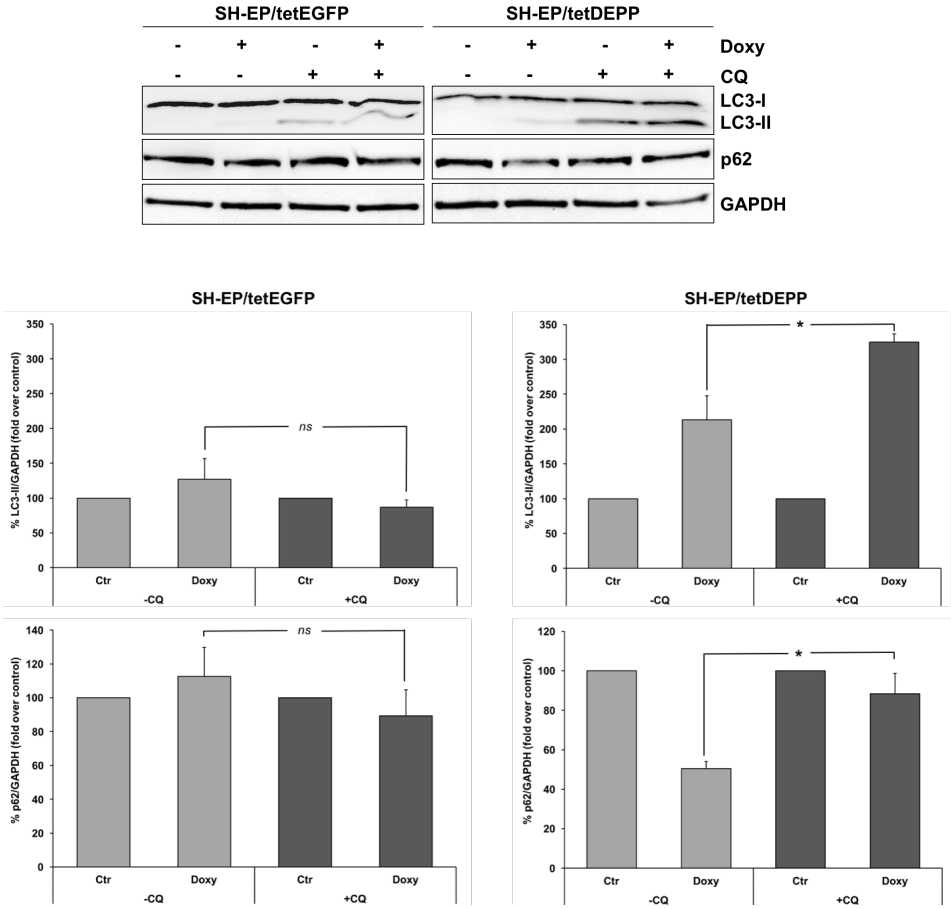

b)

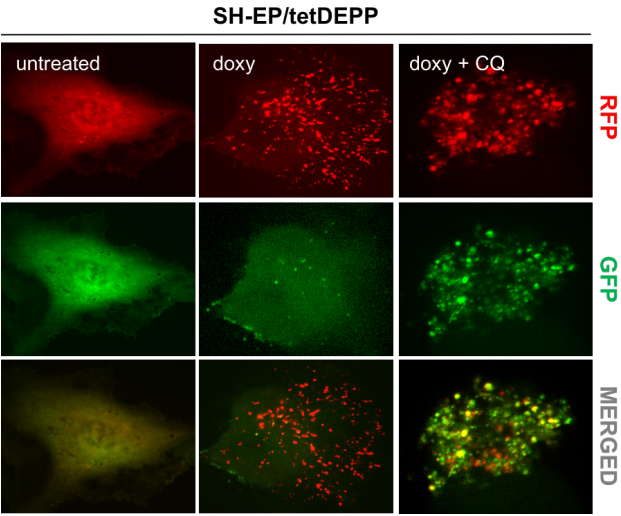

c)

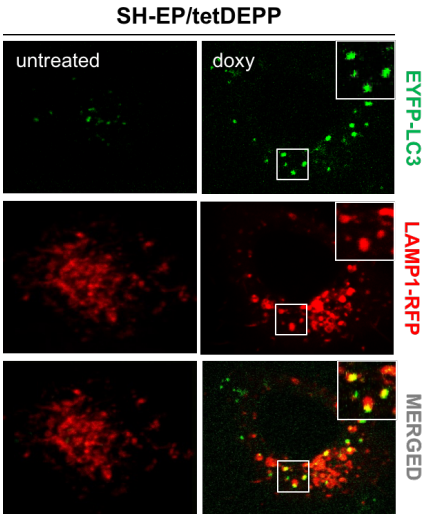

Supplement: Supplementary file 1 — DEPP expression induces autophagic flux. a SH-EP/tetEGFP and SH-EP/tetDEPP cells were treated with 200 ng/ml doxy and 100 μM CQ for 8 h. LC3-I/LC3-II and p62 expression were assessed by immunoblot analyses. GAPDH served as loading control. Densitometric analyses were performed with the ImageJ 1.48 software. Control (Ctr.) and CQ-treated cells were set as 100%. Shown are mean values ± s.e.m of three independent experiments; statistical analysis was done with the Student’s unpaired t-test, *P < 0.05. b SH-EP/tetDEPP cells were grown on ibidi μ-slide 8 well™ slides and transiently transfected with the pQCXI-Neo-DsRed-LC3-GFP plasmid. Forty-eight hours after transfection, the cells were treated with 200 ng/ml doxy and 100 μM CQ for 5 h and analyzed by confocal live-cell imaging. c SH-EP/tetDEPP cells were grown on ibidi μ-slide 8 well™ slides and transiently transfected with the pLIB-EYFP-LC3-iresPuro and the Lamp1-RFP plasmid. Forty-eight hours after transfection, the cells were treated with 200 ng/ml doxy and 100 μM CQ for 5 h and analyzed by confocal live-cell imaging. (PDF 1.54mb) [file 12943_2017_661_MOESM1_ESM.pdf]

a)

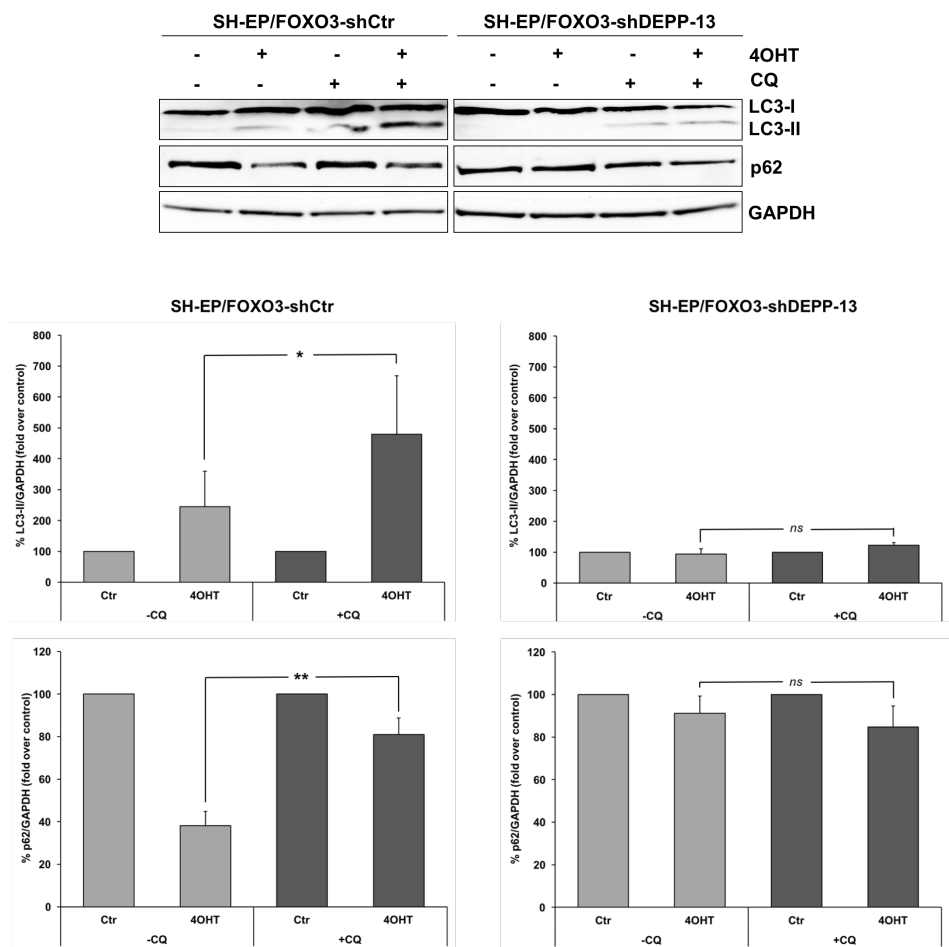

b)

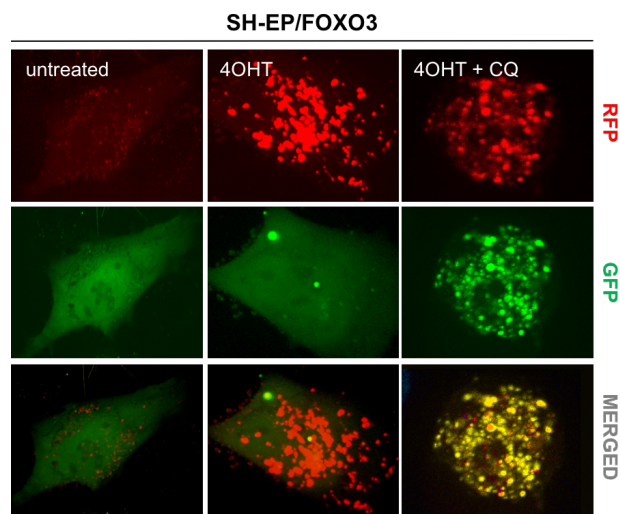

Supplement: Supplementary file 2 — FOXO3 induces autophagic flux. a SH-EP/FOXO3-shCtr and SH-EP/FOXO3-shDEPP-13 cells were treated with 50 nM 4OHT and 100 μM CQ for 8 h. LC3-I/LC3-II and p62 expression were assessed by immunoblot analyses. GAPDH served as loading control. Densitometric analyses were performed with the ImageJ 1.48 software. Control (Ctr.) and CQ-treated cells were set as 100%. Shown are mean values ± s.e.m of three independent experiments; statistical analysis was done with the Student’s unpaired t-test, *P < 0.05, **P < 0.025. b SH-EP/FOXO3 cells were grown on ibidi μ-slide 8 well™ slides and transiently transfected with the pQCXI-Neo-DsRed-LC3-GFP plasmid. Forty-eight hours after transfection the cells were treated with 50 nM 4OHT and 100 μM CQ for 5 h and analyzed by confocal live-cell imaging. (PDF 1.16mb) [file 12943_2017_661_MOESM2_ESM.pdf]

Additional file 3

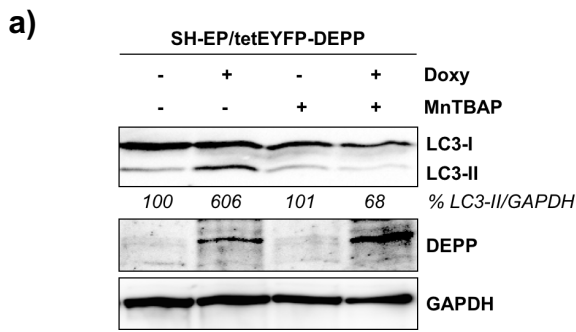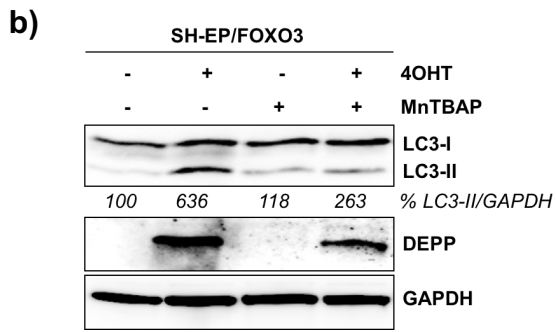

Supplement: Supplementary file 3 — FOXO3/DEPP-mediated ROS induce LC3 conversion a SH-EP/tetEYFP-DEPP cells were pretreated with 100 μM MnTBAP for one hour and incubated with 200 ng/ml doxy and 100 μM MnTBAP alone and in combination for 8 h. The LC3-I/LC3-II and DEPP expression were determined by immunoblot analyses. GAPDH served as loading control. Densitometric analyses were performed with the ImageJ 1.48 software. Untreated cells were set as 100%. b Immunoblot analyses of LC3-I/LC3-II expression of SH-EP/FOXO3 cells pretreated with 100 μM MnTBAP and incubated with 50 nM 4OHT and 100 μM MnTBAP alone or in combination for 6 h. GAPDH served as loading control. Densitometric analysis of LC3-II expression relative to GAPDH was done with the ImageJ 1.48 software. Untreated cells were set as 100%. (PDF 255kb) [file 12943_2017_661_MOESM3_ESM.pdf]

Additional file 4

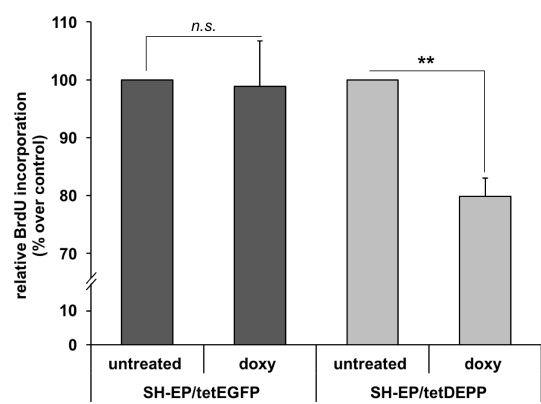

Supplement: Supplementary file 4 — Conditional expression of DEPP reduces cellular proliferation. The BrdU cell proliferation ELISA assay was used to quantify the amount of incorporated BrdU during S-phase of proliferating cells. SH-EP/tetEGFP and SH-EP/tetDEPP cells were analyzed for incorporated BrdU after treatment with 200 ng/ml doxy for 48 h. Shown are mean values ± s.e.m. of three independent experiments; statistical analysis was done with the Student’s unpaired t-test, **P < 0.025 compared to corresponding controls. (PDF 138kb) [file 12943_2017_661_MOESM4_ESM.pdf]

Additional file 5

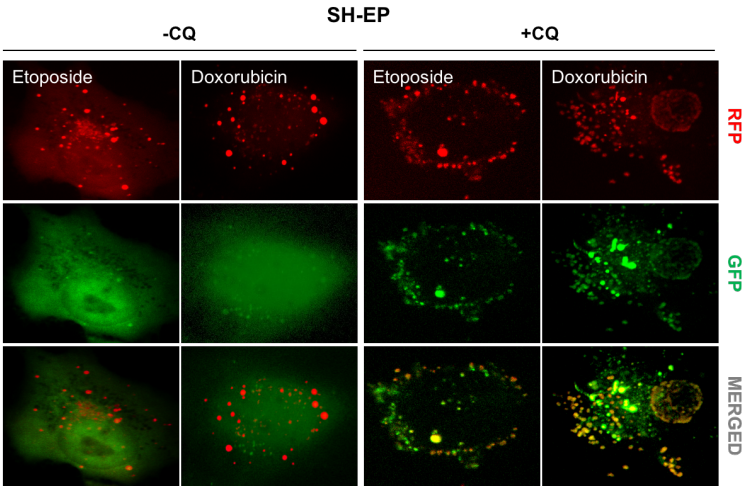

Supplement: Supplementary file 5 — Etoposide and doxorubicin induce autophagic flux in neuroblastoma cells. SH-EP cells were grown on ibidi μ-slide 8 well™ slides and transiently transfected with the pQCXI-Neo-DsRed-LC3-GFP plasmid. Forty-eight hours after transfection the cells were treated with 20 μg/ml etoposide, 0.25 μg/ml doxorubicin, and with 100 μM CQ for 6 h and analyzed by confocal live-cell imaging. (PDF 904kb) [file 12943_2017_661_MOESM5_ESM.pdf]
